# Role of the eNOS-NO System in Regulating the Antiproteinuric Effects of VEGF Receptor 2 Inhibition in Diabetes

**DOI:** 10.1155/2013/201475

**Published:** 2013-08-22

**Authors:** Andrew Advani, Kim A. Connelly, Suzanne L. Advani, Kerri Thai, Yuan Zhang, Darren J. Kelly, Richard E. Gilbert

**Affiliations:** ^1^Keenan Research Centre of the Li Ka Shing Knowledge Institute, St. Michael's Hospital, 6-151, 61 Queen Street East, Toronto, ON, Canada M5C 2T2; ^2^Department of Medicine, St. Vincent's Hospital, Melbourne, VIC 3065, Australia

## Abstract

Subtle perturbations in intraglomerular VEGF/VEGFR-2 signaling or in the influencing microenvironment can profoundly affect renal function, resulting in the apparently paradoxical observation that VEGF blockade attenuates proteinuria development in experimental diabetes despite exerting the opposite effect under other circumstances. In the present study, we sought to explore the role of eNOS-NO activity in regulating the differential response to VEGF blockade in the diabetic and nondiabetic settings. In a rodent model of accelerated renal injury, the transgenic (mRen-2)27 (Ren-2) rat, VEGFR-2 inhibition with the small molecule vandetanib resulted in an increase in urine protein excretion preceding a subsequent rise in systolic blood pressure. When compared to their normoglycaemic counterparts, diabetic Ren-2 rats exhibited an increase in the renal expression of eNOS and in urinary excretion of nitric oxide (NO) metabolites. In contrast to the heavy proteinuria observed with vandetanib in nondiabetic TGR(mRen-2)27 rats, VEGFR-2 inhibition reduced urine protein excretion in diabetic animals, despite a comparable magnitude of histological injury. However, proteinuria was markedly increased by concomitant treatment of diabetic Ren-2 rats with vandetanib and the nitric oxide synthase inhibitor L-NAME. These observations highlight the pivotal role that the eNOS-NO system plays in regulating the biologic response to VEGF within the glomerulus.

## 1. Introduction

When upregulation of vascular endothelial growth factor (VEGF) was first described in the kidneys of rats with experimental diabetes over a decade ago [1], its role in the pathogenesis of diabetic nephropathy appeared straightforward: either increased glomerular VEGF was deleterious or increased glomerular VEGF conferred compensatory renoprotection. Since that time, there has been an overwhelming expansion in our understanding of the complex role that the VEGF/VEGF receptor-2 (VEGFR-2) system may play in renal development [2], in adult glomerular homeostasis [3], and in kidney disease [4]. For instance, clinical experience of the use of anti-VEGF agents employed for their antiangiogenic effects in the oncology setting has demonstrated that blockade of VEGF signaling may occasionally result in the development of hypertension, proteinuria, or more significant renal injury [3, 5]. In contrast, a paradoxical renoprotective effect of VEGF blockade has been described in numerous studies of experimental diabetes [6–9]. Together with technological advances in the cell-specific manipulation of gene dosage, these observations have now revealed that the function of this archetypal paracrine/autocrine intraglomerular signaling network is critically affected by both subtle changes in isoform balance and subtle fluctuations in the intraglomerular milieu [10, 11]. As an illustration of dosage sensitivity of the VEGF/VEGFR-2 system, whereas pharmacological VEGF inhibition attenuates albuminuria in experimental diabetes [6–9], the opposite effect has been described following genetic VEGF obliteration [12]. In contrast to our increasing understanding of such dose/isoform effects, relatively little is known about the extrinsic factors that may influence the actions of the VEGF/VEGFR-2 system within the renal glomerulus.

One extrinsic factor that may influence the response to VEGF/VEGFR-2 signaling is the vasodilatory enzyme, endothelial nitric oxide synthase (eNOS). We recently described that the antialbuminuric effect of VEGFR-2 inhibition is negated in diabetic mice genetically deficient in eNOS [13] while, analogously, antagonism of the vasorelaxant actions of eNOS has also been shown to underlie the pressor effects of VEGF receptor blockade within the vasculature [14]. In order to determine whether altered eNOS activity may underlie the differential response to VEGF blockade with experimental diabetes, we exploited a rodent model that develops accelerated renal injury when challenged by VEGF receptor kinase inhibition, the transgenic (mRen-2)27 rat (Ren-2). The purposes of the study were the following: (1) to determine whether VEGF inhibitor-induced proteinuria may occur independently of changes within the systemic vasculature and (2) to determine whether the antiproteinuric response to VEGFR-2 inhibition in the diabetic setting is mediated through altered eNOS activity.

## 2. Materials and Methods

### 2.1. Study 1

Eight-week-old male heterozygous Ren-2 rats were randomized to receive either vehicle (polysorbate 80) (1% tween 80 Sigma) or vandetanib (25 mg/kg) (AstraZeneca, Macclesfield, UK) by daily oral gavage (*n* = 4/group). Vandetanib is a potent inhibitor of the VEGFR-2 tyrosine kinase (IC_50_ 0.04 *μ*M), with excellent selectivity versus other kinases including VEGFR-1, erbB2, MEK, CDK-2, Tie-2, IGFR-1R, PDK, PDGFR*β*, and AKT (IC_50_ range: 1.1–100 *μ*M) [15]. Animals received vehicle or vandetanib for 14 days before treatment was discontinued and animals were monitored for further 10 days. Systolic blood pressure (SBP) and urinary protein excretion were determined at baseline, day 14, and day 24. SBP was recorded in preheated rats by tail-cuff plethysmography [16]. An average SBP reading was taken from at least three consecutive recordings over a 10 min period. For estimation of urine protein excretion, rats were individually housed in metabolic cages for 24 h after 2-3 h habituation. Animals continued to have free access to tap water and standard laboratory diet during this period. After 24 h in metabolic cages, an aliquot of urine (5 mL) was collected from the 24 h urine sample and stored at −20°C for subsequent analysis, which was with the benzethonium chloride method on an Olympus analyzer.

### 2.2. Study 2

Eight-week-old male heterozygous Ren-2 rats were randomized to receive either streptozotocin (STZ) 55 mg/kg (Sigma) diluted in 0.1 M citrate buffer (pH 4.5) (diabetic, *n* = 10) or citrate buffer alone (nondiabetic, *n* = 8) by tail-vein injection after an overnight fast. Animals were monitored for 24 days before sacrifice as outlined below.

### 2.3. Study 3

Eight-week-old male heterozygous Ren-2 rats received either STZ (diabetic, *n* = 25) or citrate buffer alone (nondiabetic, *n* = 8) by tail-vein injection after an overnight fast. Diabetic Ren-2 rats were subsequently randomized to receive vehicle (polysorbate 80) (*n* = 11), 25 mg/kg vandetanib (*n* = 10) as outlined above, or 25 mg/kg vandetanib plus the NOS inhibitor *N*_*ω*_-nitro-L-arginine methyl ester (L-NAME, 20 mg/kg, Sigma) in drinking water (*n* = 4) for 24 days. 

All rats were housed in a stable environment (maintained at 22 ± 1°C with a 12 h light/dark cycle) and allowed free access to tap water and standard rat chow. Each week, rats were weighed and blood glucose was determined (AMES glucometer, Bayer Diagnostics, Melbourne, Australia). Diabetic rats received a thrice-weekly injection of insulin (2 to 4 units sc, Humulin N, Eli Lilly and Co.) to promote weight gain. SBP and urine protein excretion were determined as outlined above and glomerular filtration rate (GFR) was measured by single-shot 99m-technetium diethylenetriamine pentaacetic acid (Tc^99m^-DTPA) clearance [4]. Urinary nitrate/nitrite excretion was determined using a Nitrate/Nitrite Colorimetric Assay Kit (Cayman Chemical, Ann Arbor, MI) after housing animals in metabolic cages for 24 h as outlined above. 

At sacrifice, rats were anaesthetized with an i.p. injection of pentobarbital sodium (60 mg/kg). The left and right renal arteries were clamped and the kidneys were removed and decapsulated. The left kidney was weighed and snap frozen in liquid nitrogen before storage at −80°C for subsequent molecular biological analysis. The right kidney was sliced transversely and immersed in 10% neutral buffered formalin (NBF) for 24 h. Tissues were routinely processed, embedded in paraffin and sectioned. 

All experimental procedures adhered to the guidelines of the National Health and Medical Research Council of Australia's Code for Care and Use of Animals for Scientific Purposes and the Canadian Council on Animal Care and were approved by St. Vincent's Hospital Animal Ethics Committee, Melbourne, Australia, or by St. Michael's Hospital Animal Care Committee, Toronto, Canada. 

### 2.4. Real-Time PCR

For determination of gene expression in rat kidneys, tissue stored at −80°C was homogenized (Polytron, Kinematica Gmbh, Littau, Switzerland). Total RNA (4 *μ*g) was treated with RQ1 DNAse (1 U/*μ*L) (Promega, Madison, WI) to remove genomic DNA. RNA was reverse transcribed with a High-Capacity cDNA Reverse Transcription Kit (Applied Biosystems, Foster City, CA) according to the manufacturer's instructions. Measurement of eNOS gene expression was performed using SYBR green on an ABI Prism 7900HT Fast PCR System (Applied Biosystems). Sequence specific primers were designed to span exon-exon boundaries using Primer Express Software v1.5 (Applied Biosystems). Primers were obtained from ACGT Corp. (Toronto, ON). Primer sequences were eNOS forward GATCCTAACTTGCCTTGCATCCT, reverse TGTAATCGGTCTTGCCAGAATCC; 18S forward ATGTGGTGTTGAGGAAAGCAGAC, reverse GGATCTTGTATTGTCGTGGGTTCTG. Experiments were performed in triplicate, and data analysis was performed using Applied Biosystems Comparative C_T_ Method. 

### 2.5. Immunoblotting

Immunoblotting kidney homogenates (*n* = 7/group) was performed on nitrocellulose membranes as previously described [17] and with antibodies in the following concentrations: eNOS 1 : 1000 (Cell Signaling, Danvers, MA), phospho-eNOS Ser1177 1 : 1000 (Cell Signaling), and *β*-actin 1 : 5000 (Abcam, Cambridge, MA). After incubation with the appropriate HRP-conjugated secondary antibodies, proteins were detected by electrochemiluminescence system (GE Healthcare Life Sciences, Baie d'Urfe, QC, Canada). Densitometry was performed using Image J version 1.39. 

### 2.6. Immunohistochemistry

Immunohistochemistry for eNOS (BD Transduction Laboratories, Lexington, KY) and for the macrophage marker CD68 (ED-1 clone) (Serotec, Raleigh, NC) was performed as previously described [4, 18, 19] with the primary antibodies in the following dilutions: eNOS 1 : 400 and ED-1 1 : 100. Incubation with PBS instead of primary antiserum served as the negative control. After incubation with the appropriate horseradish peroxidase (HRP) conjugated secondary antibody, sections were labeled with Liquid Diaminobenzidine and Substrate Chromogen System (DakoCytomation) before counterstaining in Mayer's haematoxylin. For quantitation of macrophage infiltration, slides were scanned with the Aperio ScanScope System (Aperio Technologies Inc., Vista, CA) and analysed using ImageScope (Aperio). In each kidney section, the magnitude of macrophage infiltration was determined as the proportional ED-1 immunostaining in 10 randomly selected, nonoverlapping cortical fields at ×100 magnification.

### 2.7. Glomerulosclerosis Index

Eighty glomeruli were examined in PAS-stained kidney sections from each rat. The degree of sclerosis was subjectively graded on a scale of 0 to 4 as previously described [4]: grade 0, normal; grade 1, sclerotic area up to 25% (minimal); grade 2, sclerotic area 25% to 50% (moderate); grade 3, sclerotic area 50% to 75% (moderate to severe); and grade 4, sclerotic area 75% to 100% (severe). Glomerulosclerosis was defined as basement membrane thickening, mesangial hypertrophy, and capillary occlusion. A glomerulosclerosis index (GSI) was then calculated using the formula

(1) 4GSI=ΣFi(i)i=0,

where *Fi* is the percentage of glomeruli in the rat kidney with a given score (*i*). 

### 2.8. Statistics

Data are presented as means ± SEM. Statistical significance was determined by Student's *t*-test for two groups or one-way ANOVA with a Newman-Keuls post hoc comparison for more than two groups. All statistical analyses were performed using GraphPad Prism version 5.00 for Mac, GraphPad Software, San Diego, CA. A *P* value < 0.05 was regarded as statistically significant.

## 3. Results

### 3.1. VEGFR-2 Inhibitor-Induced Proteinuria Precedes the Development of Hypertension

**Figure 1:**
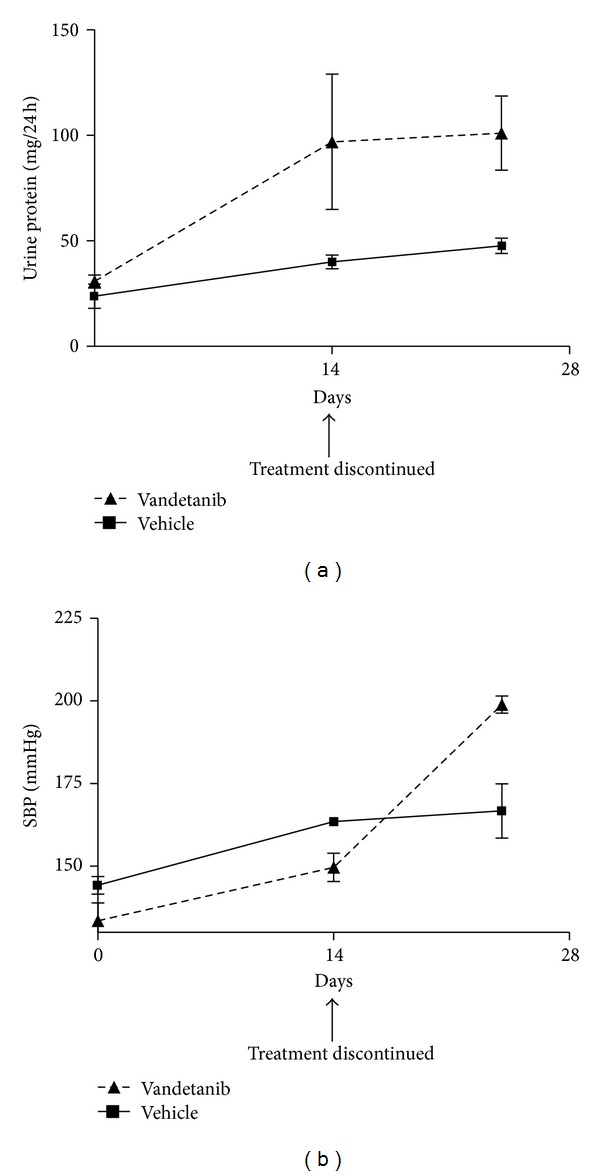
Systolic blood pressure (SBP) and proteinuria in nondiabetic Ren-2 rats treated with vehicle or vandetanib for 14 days and then observed for further 10 days. The graphs illustrate that the development of proteinuria with vandetanib precedes the rise in SBP.

To elucidate whether increased urine protein excretion occurs independently of a rise in blood pressure or whether the two are causally linked, we first treated nondiabetic Ren-2 rats with either the VEGFR-2 tyrosine kinase inhibitor vandetanib or vehicle by daily oral gavage for 14 days. At this timepoint, urine protein excretion was significantly increased in vandetanib-treated Ren-2 rats (Figure 1). Vandetanib treatment was then discontinued and animals were monitored for further 10 days. Despite cessation of vandetanib, rats previously treated with the VEGFR-2 inhibitor did not show any diminution in proteinuria 10 days after discontinuation of the drug. In contrast, systolic blood pressure (SBP) remained unchanged from its baseline after two weeks of vandetanib but rose after its discontinuation (Figure 1).

### 3.2. Diabetic Ren-2 Rats Demonstrate Increased Renal Expression of eNOS and Urinary Excretion of Nitric Oxide Metabolites

**Figure 2:**
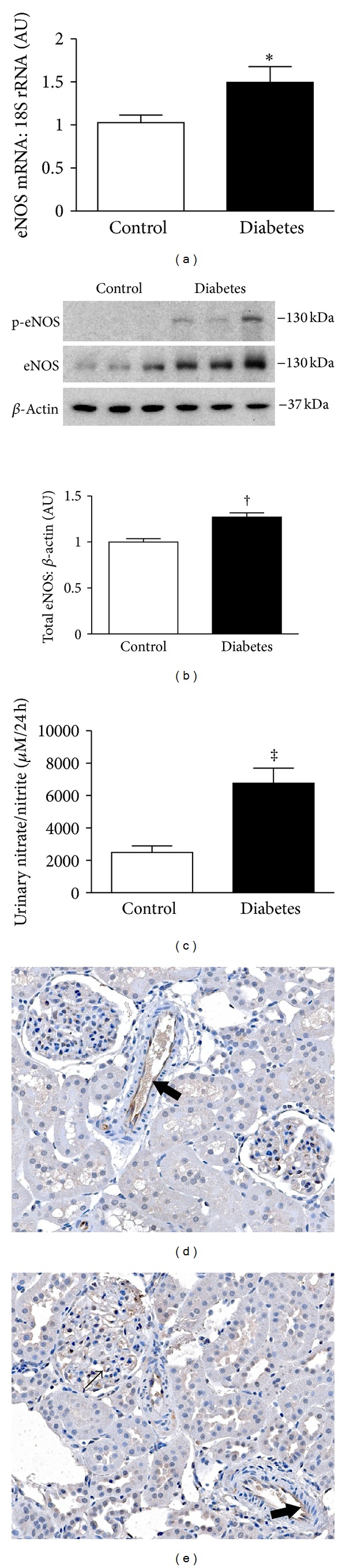
eNOS expression, activity, and urinary excretion of nitric oxide metabolites in nondiabetic (control) and diabetic Ren-2 rats at 24 days. (a) eNOS mRNA determined by real-time PCR. (b) Immunoblotting kidney homogenates from control and diabetic Ren-2 rats for total eNOS protein and eNOS Ser1177 phosphorylation (p-eNOS), indicative of enzyme activation. (c) Urinary nitrate/nitrite. ((d) and (e)) Immunohistochemistry for eNOS in kidney sections from control (d) and diabetic (e) Ren-2 rats. Original magnification ×160. AU: arbitrary units. *P < 0.05,  ^†^ *P* < 0.001, and  ^‡^ *P* < 0.01.

To examine the effect of early diabetes on eNOS expression and NO production in Ren-2 rats, animals were made diabetic with STZ and were maintained for 24 days. In comparison with nondiabetic Ren-2 rats, eNOS mRNA and protein were significantly increased in kidney homogenates of diabetic animals (Figures 2(a) and 2(b)). To confirm that increased expressed eNOS was biologically active, we immunoblotted with an antibody that binds to eNOS only when it is phosphorylated at serine residue 1177, which is indicative of enzymatic activation [20]. These experiments revealed that experimental diabetes was accompanied by a notable increase in eNOS activity (Figure 2(b)), reflected also by an increase in urinary nitrate/nitrite excretion with diabetes (Figure 2(c)). Within the kidneys of both control and diabetic Ren-2 rats, eNOS protein was restricted to the endothelial cells of arterioles and glomerular capillaries (Figures 2(d) and 2(e)). 

### 3.3. Experimental Diabetes Prevents VEGFR-2 Inhibitor-Induced Proteinuria in Ren-2 Rats

**Figure 3:**
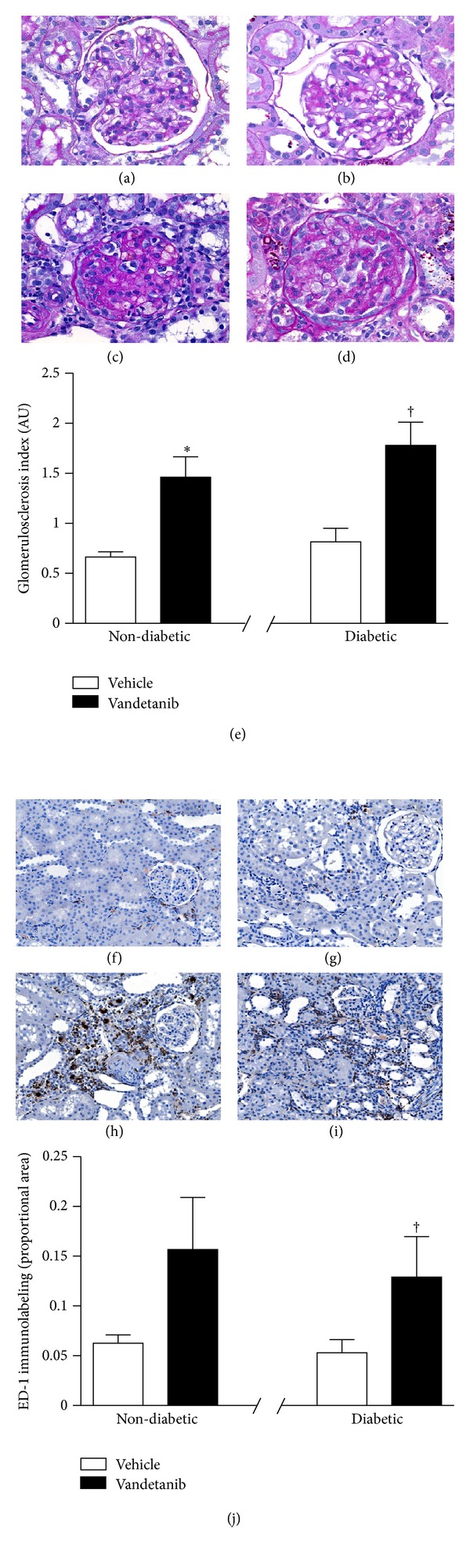
Histological changes in diabetic Ren-2 rats treated with vehicle or vandetanib for 24 days or nondiabetic Ren-2 rats treated with vandetanib for 14 days and then monitored for further 10 days. ((a)–(d)) PAS-stained kidney sections from nondiabetic Ren-2 ((a) and (c)) and diabetic Ren-2 ((b) and (d)) rats treated with vehicle ((a) and (b)) or vandetanib ((c) and (d)). Original magnification ×400. (e) Glomerulosclerosis index. ((f)–(i)) ED-1 immunolabeling in kidney sections from nondiabetic Ren-2 ((f) and (h)) and diabetic Ren-2 ((g) and (i)) rats treated with vehicle ((f) and (g)) or vandetanib ((h) and (i)). Original magnification ×160. (j) Quantitation of cortical ED-1 immunostaining. AU: arbitrary units. *P < 0.05 versus nondiabetic Ren-2 + vehicle ^†^ *P* < 0.05 versus diabetic Ren-2 + vehicle.

**Figure 4:**
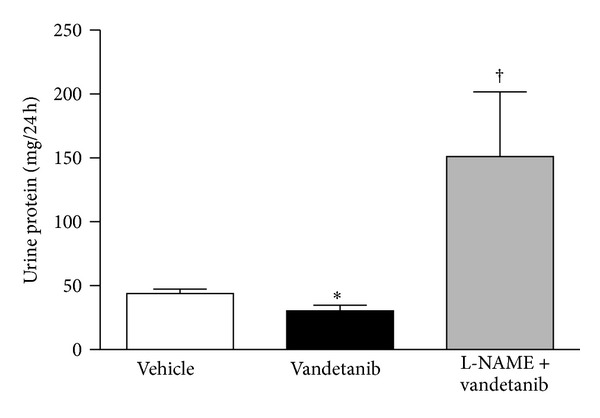
Urine protein excretion in diabetic Ren-2 rats treated with vehicle, vandetanib, or vandetanib + L-NAME for 24 days. *P < 0.05 versus vehicle and ^†^ *P* < 0.001 versus either vehicle or vandetanib.

Having demonstrated increased NO activity in the kidneys of diabetic Ren-2 rats we next went on to examine whether these animals were protected from the deleterious effects of VEGFR-2 inhibition with vandetanib. For these experiments, Ren-2 rats, were made diabetic with a tail-vein injection of STZ and were then randomized to receive either vandetanib or vehicle by once daily oral gavage for 24 days (Table 1). Vandetanib treatment resulted in an increase in the deposition of mesangial matrix in diabetic Ren-2 rats with the magnitude of injury being comparable to that seen in the nondiabetic Ren-2 rats treated with vandetanib for 14 days (Figures 3(a)–3(e)). Similarly, macrophage accumulation within the renal cortices of diabetic Ren-2 rats was increased in vandetanib treated animals, to a similar extent as that observed in nondiabetic Ren-2 rats (Figures 3(f)–3(j)). Despite an equivalent degree of renal structural injury, the presence of superimposed diabetes not only abrogated the vandetanib-induced rise in urine protein excretion observed in nondiabetic Ren-2 rats, but also actually reduced it in diabetic animals in comparison with their untreated counterparts (Figure 4). Conversely, coadministration of vandetanib with the NOS inhibitor L-NAME resulted in the development of heavy proteinuria, negating the renoprotective effect of diabetes in Ren-2 rats (Figure 4).

## 4. Discussion

The biologic actions of the VEGF/VEGFR-2 system within the glomerulus are exquisitely responsive to shifting changes in both isoform balance and extrinsic influencers. Over recent years, it has become increasingly apparent that these subtle sensitivities are responsible for the contrasting effects of VEGF antagonism observed in the diabetic and nondiabetic contexts, where VEGF antagonism augments glomerular permselectivity in the former and impairs it in the latter. In an attempt to resolve this apparent paradox, we superimposed diabetes in a rat model that develops accelerated renal injury in response to VEGF blockade. Early, experimental diabetes was associated with upregulation of the eNOS-NO system, known to be a critical regulator of the systemic effects of both VEGF inhibition and glomerular permselectivity. For the same degree of histological injury, proteinuria was attenuated in diabetic animals treated with the VEGFR-2 inhibitor vandetanib in comparison with nondiabetic animals. These observations highlight the critical role that the eNOS-NO system plays in regulating the biologic effects of VEGF/VEGFR-2 signaling within the glomerulus and the profound effects these actions may have on the renal phenotype.

Although hypertension and proteinuria may occur simultaneously in patients treated with anti-VEGF therapy, the relationship between these two adverse effects remains incompletely understood. We previously showed that, in Ren-2 rats treated with low-dose vandetanib (15 mg/kg), the rise in urine protein occurred independently of blood pressure change [4]. Similarly, glomerular injury preceded the development of hypertension in conditional podocyte-specific VEGF knockout mice [3]. In the present study, we confirmed that the renal injury associated with VEGFR-2 blockade is not a consequence of the associated hypertension. Urine protein excretion rose significantly in nondiabetic Ren-2 rats treated with vandetanib for 10 days, whereas the rise in blood pressure lagged behind. Moreover, in this accelerated model, cessation of VEGFR-2 blockade did not result in a restoration of glomerular permselectivity. The rise in systolic blood pressure in Ren-2 rats, despite discontinuation of vandetanib, suggests that transient exposure to VEGFR-2 blockade can initiate an ongoing cascade of deleterious responses under predisposing conditions. These observations are analogous to the description of nonremitting renal dysfunction and proteinuria following the discontinuation of VEGF monoclonal antibody therapy in a patient with pre-existing chronic kidney disease [21]. 

The primary source of VEGF within the kidney is the podocyte, where the growth factor crosses contrary to urinary flow and mediates its effects primarily by binding to VEGFR-2 on the surface of glomerular endothelial cells [22]. Seminal studies by Eremina et al. in 2003 demonstrated an essential role for VEGF in normal glomerular development [2]. In addition to its role in promoting vessel growth, VEGF is also one of the most potent mediators of vascular permeability known, being 50,000 times more potent than histamine on a molar basis [23]. Based on its known role in cellular growth, its upregulation in experimental diabetes, and its actions in promoting vascular permeability, de Vriese and colleagues initially hypothesized that inhibition of VEGF would prevent the onset of early renal dysfunction and first demonstrated the antialbuminuric effect of VEGF blockade in diabetes [9]. Although subsequent studies in a variety of experimental models and using a variety of pharmacological and genetic methods to block VEGF support this assumption [7, 13], more recent work in which podocyte VEGF was extinguished with the aid of an inducible Cre-loxP system has challenged this paradigm [12]. It now seems likely that both the *quantity* and the *isoform-type* of VEGF may dramatically affect glomerular permselectivity [24]. The influence of the eNOS-NO system on the VEGF response also likely exhibits a dose effect. For instance, in the present study VEGFR-2 blockade marginally, but non-significantly, attenuated hyperfiltration in diabetic Ren-2 rats that exhibited increased renal eNOS-NO activity, whereas we previously found GFR to be markedly reduced with vandetanib in normoglycemic Ren-2 rats [4]. 

The antiproteinuric effect of VEGF blockade in some rodent models of diabetes, but not others [25], suggests that, along with intrinsic characteristics of the VEGF/VEGFR-2 signaling network, of parallel importance is the influence of extrinsic influencing factors affected by the metabolic and hemodynamic perturbations of diabetes. One of the early features of experimental diabetic nephropathy is upregulation of the eNOS-NO system [26]. The role that eNOS-derived NO plays in the pathogenesis of kidney disease in diabetes is complex. While uncoupling of eNOS in the face of substrate deficiency may contribute to oxidative stress in diabetes [17], deletion of the eNOS gene predisposes diabetic mice to develop heavy albuminuria [27–30]. In Ren-2 rats, as in other models, we observed that eNOS expression, activity, and NO metabolite excretion were increased in the setting of early experimental diabetes. This eNOS upregulation was associated with a profound attenuation of the proteinuria that occurred with VEGFR-2 blockade, to such an extent that vandetanib-treated diabetic rats had significantly lower urinary protein excretion than their vehicle-treated counterparts. The divergent effects of VEGFR-2 inhibition on urine protein excretion under control and diabetic conditions, despite a comparable magnitude of histological injury, suggest that whereas VEGF may exert biologic effects along the length of the nephron [31], interaction between the VEGF and eNOS systems plays a particularly important role at the level of the filtration barrier. Analogously, the induction of diabetes in eNOS deficient mice results in a podocytopathy and rapid increase in albumin excretion in the absence of light microscopic features of additional renal injury, with the rise in albuminuria being unresponsive to VEGFR-2 blockade [13]. 

Numerous studies point to a critical role for NO in mediating the effects of VEGF on angiogenesis, vascular permeability, and blood pressure regulation [14, 32–34]. For instance, the blood pressure difference between mice treated with a VEGFR-2 monoclonal antibody and those receiving vehicle was negated with concomitant administration of the NOS inhibitor, L-NAME [14]. The present study extends these observations and suggests that the antiproteinuric effects of VEGFR-2 blockade in diabetes are also likely to be mediated by altered NO activity. Moreover, increased renal expression of eNOS in models of early, experimental diabetes may potentially underlie the previously described “VEGF paradox” of diabetic nephropathy [35–37]. 

## 5. Conclusions

In conclusion, the findings of the present study illustrate the profound influence that the extrinsic microenvironment may have on the biologic effects of the VEGF/VEGFR-2 system, where diabetes-induced eNOS expression modulates the response to VEGF blockade. Whether a similar scenario also exists in humans undergoing treatment with such agents remains to be determined.

## Figures and Tables

**Table 1:** Metabolic parameters for diabetic Ren-2 rats treated with vehicle or vandetanib.

	Vehicle	Vandetanib
Body weight (g)	336 ± 10	296 ± 15
Blood glucose (mmol/L)	27.6 ± 1.2	27.1 ± 1.3
Systolic BP (mmHg)	205 ± 10	224 ± 13
GFR (mL/min/kg)	16.7 ± 0.7	15.2 ± 0.5
